# Radiotherapy for keratoacanthoma of facial skin: A case report and review of literature

**DOI:** 10.3389/fonc.2022.1032090

**Published:** 2023-01-09

**Authors:** Xiaojing Jia, Ying Ge, Hongyong Wang, Yan Ma

**Affiliations:** Department of Radiation Oncology, The Second Hospital of Jilin University, Changchun, China

**Keywords:** radiotherapy, intensity modulated radiotherapy, superficial electron beam, keratoacanthoma, case report

## Abstract

**Background:**

Keratoacanthoma (KA) is a benign tumor that arises from the infundibulum of hair follicles. However, some researchers believe that KA is a subtype of squamous cell carcinoma (SCC) or a borderline tumor. There are two types of KA: single-type and multiple-type. Surgical resection is the first-line treatment for KA. The treatment options for patients with large lesions who are not surgical candidates are limited. We present a case of single-type KA patients with basic diseases and large lesions that were untreatable surgically, but the lesions essentially disappeared after radiotherapy. No recurrences were discovered during the two-year follow-up.

**Case Description:**

A 62-year-old male patient was admitted to the dermatology department of our hospital in June 2020 due to the discovery of a red papule on the right face two months prior, with occasional itching, which increased gradually. Pathological examination confirmed the diagnosis of KA. Due to the large lesions and underlying diseases, he was transferred to our radiotherapy department for radiotherapy after consultation. Since the surface of the lesion is uneven and close to the corner of the eye, we adopted intensity modulated radiation therapy (IMRT) at the beginning of radiotherapy. Following the reduction of the lesion, superficial electron beam and added a bolus with thickness of 5mm on the surface of the lesion was continued. The target dose: 42Gy/21 fractions (6MV X-ray, 22Gy; 2Gy/fraction; a total of 11 fractions, 6MeV electron beam, 20Gy; 2Gy/fraction; a total of 10 fractions). By the end of radiotherapy, the patient’s facial tumor was dry and subsided. The facial tumor subsided significantly two years after radiotherapy, and the damaged skin on the face recovered to a flat shape.

**Conclusions:**

The treatment experience of this case shows that IMRT combined with superficial electron beam radiotherapy may be an effective treatment for single-type KA patients with basic diseases and large lesions that are not suitable for surgery, and it is worth further study.

## Introduction

Hutchinson first reported Keratoacanthoma (KA) in 1889. KA is also known as self-healing cutaneous squamous cell carcinoma, pseudotumor, and molluscum contagiosum due to its various pathological conditions. Although initial reports of KA have been around for a century, its etiology, epidemiology, pathology, pathogenesis, prognosis, and treatment remain unknown. Surgery is the primary treatment for KA; however, radiotherapy alone is rarely reported. This article describes the treatment of a patient with facial KA with radiotherapy. The patient had underlying diseases such as cerebral infarction and diabetes, and the lesion was relatively large. Following anti-infection and other symptomatic treatments, the patient was transferred from the dermatology department to our department for radiotherapy, which achieved a good curative effect. The following describes the diagnosis and treatment process.

## Case presentation

**Figure 1:**
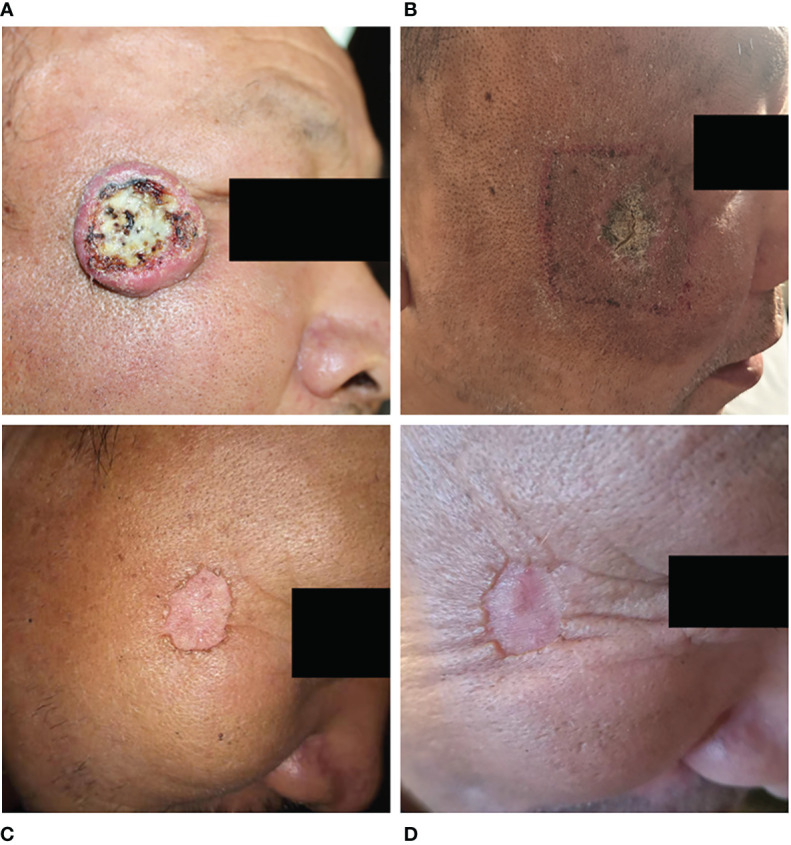
Change in facial lesions before and after radiotherapy. **(A)**, The facial lesions appeared as “crater-like” ulcers before the treatment; **(B)**, By the end of radiotherapy, the facial lesions regressed significantly and formed dry scabs; **(C)**, One year after radiotherapy, the facial lesions basically disappeared; **(D)**, Two years after radiotherapy, the edge of the lesions flattened compared to the first year.

**Figure 2:**
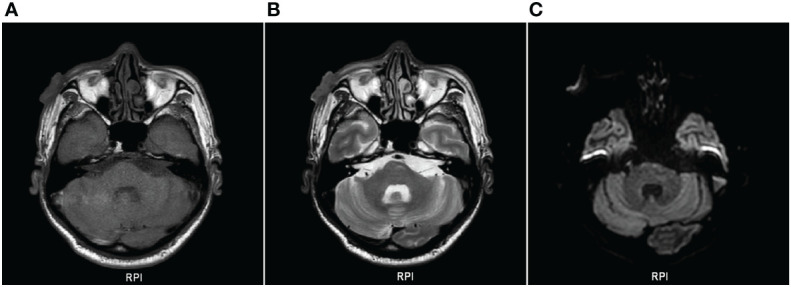
Plain scan of superficial soft tissue on MRI. **(A)**, T1WI showing slightly hyperintensity; **(B)**, T2WI showing slightly hyperintensity; **(C)**, DWI and brain parenchyma were isointense.

**Figure 3:**
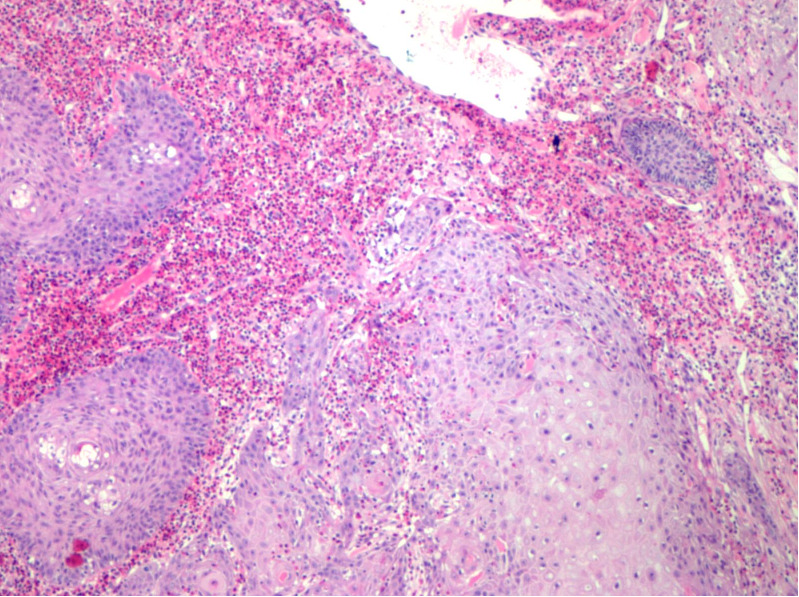
Light microscopic findings: atypical hyperplasia of squamous epithelium with suppurative inflammation.

In June 2020, a 62-year-old male patient presented to the Department of Dermatology of our hospital because a red papule was discovered on the right face two months prior, with occasional itching that gradually increased. He had a 7-year history of diabetes, underwent foot surgery due to traffic accident in April 2020, and had a 4-year history of cerebral infarction. Physical examination after admission: a “crater”-like ulcer was found on the skin of the right face, with a size of about 3.5 × 3.5 cm, protruding from the body surface, the surface was uneven and hard, and the formation of keratin plugs was seen in the middle of the ulcer, accompanied by ulceration, exudation, crusting, and a clear boundary, accompanied by pain and itching, without any swollen lymph nodes around (**Figure 1A**). Auxiliary examination on admission: An MRI superficial soft tissue plain scan revealed that local soft tissue shadows on the right side of the face had thickened, and localized lumps had grown outward. Heterogeneous, with a somewhat high signal on T2WI and a slightly high signal on T1WI, the lesion grew medially and lacked a distinct border between adjacent muscles. There was no abnormal signal in the surrounding bone, but the soft tissue of the right eyelid had thickened. DWI and brain parenchyma was isointense. Diagnosis prompt: right facial mass (**Figure 2**). Chest computed tomography (CT) revealed bronchitis and emphysema in both lungs. Right middle lobe and bilateral lower lobe inflammation, inflammatory cord, thoracic aorta, and coronary arteriosclerosis were observed. Color Doppler ultrasound of the neck showed lymph node-like echoes around the bilateral submandibular region. The border between the cortex and pulp was still visible. Ultrasound diagnosis: the lymph nodes surrounding the submandibular area on the left side were visible, and the lymph nodes around the submandibular area on the right side were slightly larger. Laboratory tests, including routine blood tests and liver and kidney function tests, revealed no obvious abnormalities. Tumor marker levels were within normal limits. The pathological findings indicated that the changes were consistent with keratoacanthoma (**Figure 3**). Diagnosis: Keratoacanthoma, a skin infection.

**Figure 4:**
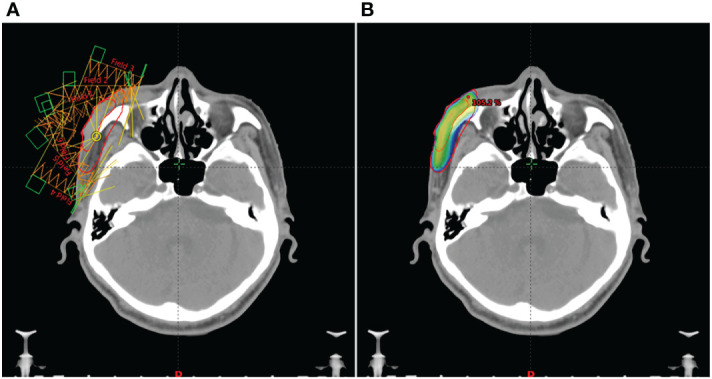
The patient’s IMRT treatment plan. **(A)**, In order to make each part of the lesion receive uniform irradiation, we used IMRT treatment plan with 6 radiation fields. **(B)**, The red line on the outer layer represents the area to be irradiated, and the gradient color image from blue to red represents the 100% prescription dose range. It can be seen that the two are highly consistent.

**Figure 5:**
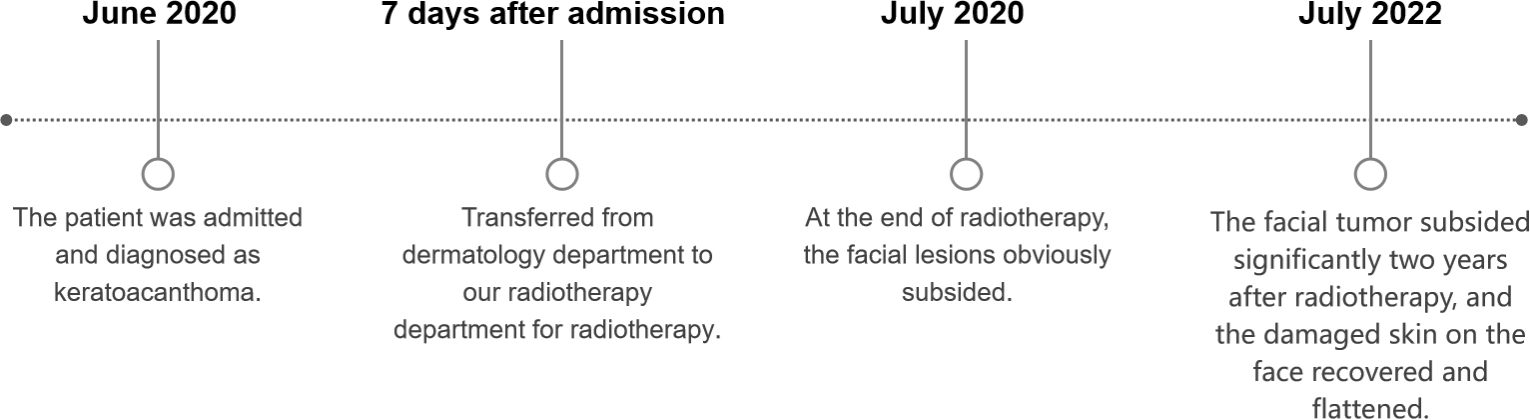
Some important time points for the treatment and follow-up of the patient after admission.

After admission, the patient obtained swabs from the lesions in the dermatology department for bacterial and fungal culture and drug sensitivity test, and the results showed that there was golden grape ball infection. Tobramycin was given to the patient and applied externally to the lesion. At the same time, the patients were given recombinant human epidermal growth factor and sprayed on the lesion for external use to promote skin repair. In addition, the patient has bronchitis and pulmonary infection. After consultation in the respiratory department, the patients were given 1.0g cefotaxime twice a day for intravenous infusion. Because the patient has a history of cerebral infarction, troxerutin and cerebroprotein hydrolysate injection 10ml, once a day, intravenous infusion. The endocrinology department was also invited to regulate the blood glucose level of the patient. After symptomatic treatment, the lesion did not subside. Considering that it is difficult to predict the maximum size of the lesion in this patient before regression or how it will eventually heal. There is a risk of disease expansion and canceration, so early intervention is required. Because the large lesions are located near the corner of the eye, the surgical treatment is difficult, and there are cosmetic problems after surgery. The patient has a history of diabetes, and the surgical wound is difficult to heal. In addition, the patient has other basic diseases such as cerebral infarction and emphysema. At the same time, the patients and their families also refused to undergo surgical treatment. After multi-disciplinary consultation, we chose radiotherapy for the patient. After 7 days of treatment in dermatology department, he was transferred to our radiotherapy department for radiotherapy. Radiotherapy for facial tumors was administered after ensuring that there were no contraindications. According to the patient’s condition, 6 MV X-ray was used for intensity modulated radiotherapy (IMRT) at the beginning of treatment(**Figure 4**). When the number of radiotherapy reaches 11 fractions, the disease subsides obviously. Since then, we have used superficial electron beam and added a bolus with thickness of 5mm on the surface of the lesion for radiotherapy. The target dose: 42Gy/21 fractions (6MV X-ray, 22Gy; 2Gy/fraction; a total of 11 fractions, 6MeV electron beam, 20Gy; 2Gy/fraction; a total of 10 fractions). The radiotherapy proceeded smoothly. There was first-degree radiation skin damage in the irradiation field during the application of electron beam irradiation. We gave the patient recombinant human epidermal growth factor for external use to promote the repair of damaged skin. There were no obvious abnormalities in laboratory tests, such as routine blood tests, during radiotherapy. The whole radiotherapy process lasted for 1 month. The patient’s facial tumor was dry and subsided by the end of radiotherapy (**Figure 1B**). The facial tumor subsided significantly two years after radiotherapy, and the damaged skin on the face recovered and flattened (**Figures 1C, D**). **Figure 5** summarizes the important events of this patient according to the timeline.

## Discussion

KA is currently thought to be a benign tumor originating from the infundibulum of hair follicles. Some researchers believe that KA is a subtype of squamous cell carcinoma (SCC) or a borderline tumor. The typical histopathological features of KA are a well-circumscribed “crater-like” tumor raised in the skin, symmetrical in structure, filled with a keratin plug in the center, and an upper part of the tumor mainly composed of large, pale keratinocytes. The proliferating keratinocytes were pale and transparent with abundant cytoplasm and light staining of eosinophilic ground-glass-like cells. In some areas, the cells were atypical, with a pathological mitotic phase and inflammatory cell infiltration in the dermis. As the pathological features of KA are very similar to those of squamous cell carcinoma (SCC), the immunohistochemical examination cannot strictly distinguish between the two, so the current KA controversy is primarily focused on the pathological features of KA and SCC. Adequate pathological sampling is required when performing the pathological biopsy to ensure the distinction between KA and SCC. Researchers have distinguished KA from SCC at the gene lineage level (1). To improve the treatment methods, the identification of KA and SCC should be the focus of the research.

KA has two typical characteristics compared with other neoplastic lesions. One is that KA exhibits the typical “crater”-like ulcers with rapid growth and spontaneous regression, and the other is that KA has a potential benign-malignant borderline. Pathological features, that is, a small number of KAs have recurrence or even canceration after resection or spontaneous regression. The true incidence of KA may be underestimated because clinicians misdiagnose KA as SCC and there are fewer reports on KA. Furthermore, some patients recovered before presentation; therefore, they were not adequately classified. KA is more common in men and primarily affects middle-aged and older adults. The incidence rate is positively related to age, with the peak age of onset occurring between 65 and 71 years (2, 3). Ultraviolet radiation, trauma, human papillomavirus infection, immunosuppressive agents, and genetic factors may be the causes of KA (4–6). KA is most commonly seen in the skin of middle-aged and elderly patients exposed to ultraviolet light for a long time, such as the scalp, nose, cheeks, around the eyes, lips, upper limbs, and so on, with a few cases occurring in the conjunctiva, oral mucosa, legs, female genitals (7–9). There are two types of KA: single-type and multiple-type, with single-type KA having the highest incidence (10). Single-type is prevalent among middle-aged and elderly men exposed to ultraviolet radiation for a long time. The initial onset is usually small red papules with occasional itching symptoms, and it rapidly grows into a hemispherical nodule with a crater-like depression in the center within 6 - 8 weeks. Inside, white keratinous plugs develop, and a “lip-like” epidermis protrudes from the skin around them. The diameter of hemispherical nodules is usually approximately 1 - 2 cm, and some can reach more than 20 cm. Some KAs can reach more than 20cm, also known as marginal centrifugal keratoacanthoma marginatum (KCM), which belongs to the variant type of giant KA. It is characterized by that giant centrifugal nodules usually do not have a typical “crater like” shape, lesions usually grow centrifugally, present a “coral reef like” appearance, and generally do not have a self-healing tendency. Miedzinski first described KCM in 1962, and the patient’s disease site was on the back of the hand (11). Except for KCM, the duration of single-type lesions is typically 2 - 12 months, with some patients experiencing lesions lasting longer than a year. Multiple-type KA is rare and more common in older women who have had long-term exposure to UV light on the skin of their legs. Multiple-type KA is divided into two subtypes: Multiple self-healing squamous epitheliomas (MSHSE) and generalized eruptive keratoacanthoma (GEKA). Among them, MSHSE has a certain familial aggregation tendency. Goudie et al. suggested that its occurrence could be caused by mutations in the TGFβ1 gene, but some studies have suggested that the pathogenesis of the disease could be caused by multiple gene mutations (12, 13). MSHSE has an earlier age of onset and a slower onset than GEKA, usually subsides within a few months, and can continue to appear anywhere on the body surface, usually as large papules (approximately 1 cm on the face, larger on the limbs), with keratin plugs, shedding ulcers. GEKA is usually sporadic and does not tend to familial aggregation. It is mostly adult-onset, and its onset is more acute than MSHSE. The majority of skin lesions are follicular papules that range from 1 - 2 mm and can number in the hundreds or thousands. The patient, in this case, is an elderly male. Tumor lesions appeared on the face that had been exposed to external beams for a long time. His facial KA had the typical clinical features of single-type KA, showing a “crater-like” morphology.

KA demonstrated self-healing abilities. Conservative observation of disease progression can be used as a tool. However, waiting for observation may result in the loss of the best treatment option for KA, with a wide range of lesions and continuous and rapid growth. It is particularly critical to select the most appropriate treatment method. The treatment of KA includes surgery, chemotherapy, photodynamic therapy, and so on. Surgical resection is the first choice for the treatment of single-type KA. There is no uniform standard for the extent of lesion resection. A negative margin indicated that the lesion had been completely removed, whereas a positive margin did not indicate recurrence (14). In addition, for patients who are not suitable for surgery due to large lesions, preoperative neoadjuvant chemotherapy can be used to reduce the size of the lesions before surgery. Martorell et al. conducted a prospective randomized clinical trial. Patients with 1.5 cm lesions were randomly assigned to one of two groups: preoperative methotrexate neoadjuvant chemotherapy group (n= 10) or simple surgery (n = 15). However, only one patient in the surgical treatment group shrank the lesion slightly, and neither group experienced any complications during treatment. This study demonstrated the safety and efficacy of preoperative neoadjuvant chemotherapy (15). In addition, for patients with massive lesions and whose own factors are not suitable for surgery, radiotherapy can be used to control the development of lesions. As most KA patients receive surgical treatment, there are few reports on radiotherapy for KA, and the treatment experience is relatively insufficient (16). At present, there is no uniform standard for the total radiation dose, fractional dose and time of radiotherapy for most borderline tumors, which are mostly determined according to clinical experience. The treatment principle is that the radiation dose should be less rather than more, but the radiation dose should fully control the growth of the lesion and make it disappear to prevent recurrence. Usually, the radiation dose required for skin malignant tumors is 50-70Gy, while the radiotherapy dose for skin borderline tumors or benign skin lesions should be lower than that for skin malignant tumors. In this study, We gave the patient a radiation dose of 42Gy and used different radiotherapy techniques according to the location and shape of the lesion. Because the patient has a large lesion, it is located near the corner of the eye, protruding from the body surface, and the lesion surface is uneven. In order to get better treatment for the lesion, we used 6 MV X-ray for IMRT at the beginning of the treatment. IMRT can obtain a highly conformal three-dimensional dose distribution in the target area by adjusting the radiation dose of different radiation fields, so as to increase the dose in the target area and improve the treatment gain ratio without increasing or even reducing the radiation dose of the surrounding normal tissues. Through this radiotherapy technology, each part of the lesion is guaranteed to receive uniform radiation (**Figure 4**). When we worked out the IMRT treatment plan for this patient, we took into account that the morphology of the patient’s lesion was more complex, like a volcanic crater. At the same time, there is infection, and covering the surface of the lesion with a bolus may cause the lesion to bleed. Therefore, we did not apply bolus in IMRT radiotherapy for this patient. During the superficial electron beam treatment, the lesion on the skin surface were basically recovered. In order to increase the radiotherapy dose on the skin surface, we applied a bolus with a thickness of 5 mm. During radiotherapy, the lesion subsided rapidly. When the radiation dose reached 22 Gy, the lesion subsided significantly and almost became flat. Since then, we have used superficial electron beam for radiotherapy. When the prescribed radiation dose of 42Gy was reached, the lesion almost disappeared. Subsequently, we had a close follow-up, and there was no recurrence after 2 years of follow-up.

The treatment of this case shows that IMRT combined with superficial electron beam therapy may be a better treatment option for KA patients who are not suitable for surgery, and it is worth further study. Although KA is a self-healing disease, a recurrence or malignant change is still possible. Clinically, follow-up after KA treatment should be performed to ensure that a small number of KA patients receive timely and effective treatment, even if they have recurrence or malignant transformation. The limitation of the present study is that we did not use 3D printed boluses when conducting radiotherapy for patients with superficial electron beam. The research of Wang X et al. shows that 3D printed boluses can reduce the air gap between skin surface and bolus, improve the accuracy and uniformity of radiation dose, better protect normal tissues, and have obvious advantages in cost and time efficiency (17). In the course of radiotherapy for such patients in the future, 3D printed boluses can be considered to achieve better therapeutic effect.

## Data availability statement

The original contributions presented in the study are included in the article/supplementary material. Further inquiries can be directed to the corresponding author.

## Ethics statement

The studies involving human participants were reviewed and approved by The second hospital of Jilin university. The patients/participants provided their written informed consent to participate in this study. Written informed consent was obtained from the individual(s) for the publication of any identifiable images or data included in this article.

## Author contributions

Conceptualization: YM. Investigation and data collection: YG and HW. Writing - review & editing: XJ, YM and YG.

## References

[1] Ra SH Su A Li X Zhou J Cochran AJ Kulkarni RP. Keratoacanthoma and squamous cell carcinoma are distinct from a molecular perspective. Mod Pathol (2015) 28(6):799–806. doi: 10.1038/modpathol.2015.5 25676557

[2] Schwartz RA. Keratoacanthoma: a clinico-pathologic enigma. Dermatol Surg (2004) 30(2):326–33. doi: 10.1097/00042728-200402002-00015 14871228

[3] Vergilis-Kalner IJ Kriseman Y Goldberg LH. Keratoacanthomas: Overview and comparison between Houston and minneapolis experiences. J Drugs Dermatol (2010) 9(2):117–21. 20214172

[4] Sullivan JJ. Keratoacanthoma: the Australian experience. Australas J Dermatol (1997) 38 Suppl 1:S36–39. doi: 10.1111/j.1440-0960.1997.tb01007.x 10994470

[5] Dufresne RG Marrero GM Robinson-Bostom L. Seasonal presentation of keratoacanthomas in Rhode island. Br J Dermatol (1997) 136(2):227–9. doi: 10.1111/j.1365-2133.1997.tb14901.x 9068737

[6] Karaa A Khachemoune A. Keratoacanthoma: a tumor in search of a classification. Int J Dermatol (2007) 46(7):671–8. doi: 10.1111/j.1365-4632.2007.03260.x 17614793

[7] Ozkan F Bilgiç R Cesur S. Vulvar keratoacanthoma. APMIS (2006) 114(7–8):562–5. doi: 10.1111/j.1600-0463.2006.apm_326.x 16907863

[8] Svirsky JA Freedman PD Lumerman H. Solitary intraoral keratoacanthoma. Oral Surg Oral Med Oral Pathol (1977) 43(1):116–22. doi: 10.1016/0030-4220(77)90360-7 264336

[9] Szabo BA Şovrea AS Bartoş DM Bartoş A Georgiu C. Keratoacanthoma of the conjunctiva - case report and review of the literature. Rom J Morphol Embryol (2017) (4):1605–9. 29556663

[10] Kwiek B Schwartz RA. Keratoacanthoma (KA): An update and review. J Am Acad Dermatol (2016) 74(6):1220–33. doi: 10.1016/j.jaad.2015.11.033 26853179

[11] Miedzinski F Kozakiewicz J. Keratoacanthoma centrifugum–a special variety of keratoacanthoma. Hautarzt (1962) 13:348–52. 14473569

[12] Goudie DR D'Alessandro M Merriman B Lee H Szeverényi I Avery S. Multiple self-healing squamous epithelioma is caused by a disease-specific spectrum of mutations in TGFBR1. Nat Genet (2011) 43(4):365–9. doi: 10.1038/ng.780 21358634

[13] Kang HC Quigley DA Kim IJ Wakabayashi Y Ferguson-Smith MA D’Alessandro M. Multiple self-healing squamous epithelioma (MSSE): Rare variants in an adjacent region of chromosome 9q22.3 to known TGFBR1 mutations suggest a digenic or multilocus etiology. J Invest Dermatol (2013) 133(7):1907–10. 10.1038/jid.2013.45 PMC3664264 23358096

[14] Jackson JE Kelly B Petitt M Uchida T Wagner RF Jr. Predictive value of margins in diagnostic biopsies of nonmelanoma skin cancers. J Am Acad Dermatol (2012) 67(1):122–7. doi: 10.1016/j.jaad.2011.09.027 22088427

[15] Martorell-Calatayud A Requena C Nagore E Sanmartín O Serra-Guillén C Botella-Estrada R. Intralesional infusion of methotrexate as neoadjuvant therapy improves the cosmetic and functional results of surgery to treat keratoacanthoma: results of a randomized trial. Actas Dermosifiliogr (2011) 102(8):605–15. doi: 10.1016/j.ad.2011.03.013 21742301

[16] Matsumura Y Honda R Ito S. Ineffectiveness of radiotherapy for the treatment of keratoacanthoma centrifugum marginatum: A case report. Dermatol Ther (2019) 32(4):e12920. doi: 10.1111/dth.12920 30977219

[17] Wang X Wang X Xiang Z Zeng Y Liu F Shao B. The clinical application of 3D-printed boluses in superficial tumor radiotherapy. Front Oncol (2021) 11:698773. doi: 10.3389/fonc.2021.698773 34490095 PMC8416990

